# Recent advances in nano scaffolds for bone repair

**DOI:** 10.1038/boneres.2016.50

**Published:** 2016-12-13

**Authors:** Huan Yi, Fawad Ur Rehman, Chunqiu Zhao, Bin Liu, Nongyue He

**Affiliations:** 1 State Key Laboratory of Bioelectronics (Chien-Shiung Wu Lab), School of Biological Science and Medical Engineering, Southeast University, Nanjing, China; 2 Department of Biomedical Engineering, School of Basic Medical Sciences, Nanjing Medical University, Nanjing, China; 3 Hunan Key Laboratory of Green Chemistry and Application of Biological Nanotechnology, Hunan University of Technology, Zhuzhou, China

## Abstract

Biomedical applications of nanomaterials are exponentially increasing every year due to analogy to various cell receptors, ligands, structural proteins, and genetic materials (that is, DNA). In bone tissue, nanoscale materials can provide scaffold for excellent tissue repair via mechanical stimulation, releasing of various loaded drugs and mediators, 3D scaffold for cell growth and differentiation of bone marrow stem cells to osteocytes. This review will therefore highlight recent advancements on tissue and nanoscale materials interaction.

## Introduction

Bone or osseous tissue is a significant and dynamic supporting connective tissue that continues to remodel and rebuild throughout the lifetime of an individual. Since bone is a scaffold of the body that is responsible for support, protection, locomotion and load bearing. In addition, it also undertakes responsibility for hematopoiesis, mineral homeostasis and other functions.

Currently, musculoskeletal maladies that result in tissue degeneration and inflammation are the main reasons for the disability and associated diseases around the globe.^1^ In 2013, as reported in the Global Burden of Disease Study 2013 (GBD 2013) led by the Institute for Health Metrics and Evaluation (IHME), the burden caused by musculoskeletal maladies around the globe was 149 435 700 disability-adjusted life-years (DALYs), that mainly included rheumatoid arthritis, osteoarthritis, gout, low back and neck pain and other musculoskeletal disorders.^2^ DALY is a unit to express the health losses from a type of disease and injury which computes the years of living with disability and years of life lost.^3^ Even though bone tissue has internal repair and regeneration capacity, healing of large-scale bone defects caused by trauma, infection and tumor still needs external interventions.^4^ There is, therefore, a huge demand for technologies and materials to ameliorate such kind of maladies.

Nanomaterials are synthetic or natural materials that have less than 100 nm size in either direction.^5^ Technically, any material at nanoscale can be regarded as nanomaterial, but for better biomedical applications, the size should be in the range of 10–100 nm. Size above 100 nm may induce embolism and can be phagocytized and removed by the spleen, whereas reticulo-endothelial system and kidneys can readily clear the materials with size less than 10 nm.^6^ Moreover, the size below 10 nm is more toxic and reactive due to higher surface density and increased surface reactive electrons. Nano-biomaterials are structural analogs to various body proteins, receptors, ligands and DNA (typically 5–20 nm size). This allows them to interact freely with various body receptors, easily crossing the cell membrane.^7^ Nano-biomaterials are widely used in gene therapies,^8^ nano-drug delivery systems,^9^ cancer and various other disease theranostics,^10–14^ sono and photo-dynamic therapies,^15^ prosthetic orthopedic implants,^16^ tissue engineering,^17^ and so on. Nano-biomaterials combined with other medical methods may therefore have a key role in the near future.^18^

In this review, we mainly focus on the recent advances in nanomaterials application for bone tissue repair and prosthetic implants used to support the skeletal system. Conventional biomaterials that are used for bone tissue amelioration have been reported to have complications that result in elevated implant failure rate and delayed bone reparation. In this review, we will focus on the recent advances in nanotechnology for bone and supporting tissues reparation and amelioration.

## Bone biology and regeneration

Macroscopically, bone tissue evolves into a variety of appearances to support different functions. To simplify the system, bones can be classified based on shape (that is, long, short, flat, and irregular bone), location (that is, axial and appendicular bone), or composition (that is, compact or spongy bone), and so on. Depending on their function, bones are only different in the pattern of arrangement, though they are all composed of same materials.

### The bone matrix

Bone matrix consists of organic component, inorganic mineral component and water. Organic component accounts for approximately 25% of the weight of bone matrix, which includes type I collagen (~90%) and other non-collagenous proteins (for example, sialoprotein and osteopontin).^19^ The non-collagenous proteins and proteoglycans account for a small total weight of organic component, though they still have an important role in osteoblast differentiation and tissue mineralization.^20^ The mineral compartment of bone contributes to ~65% of the bone matrix by weight (primarily in the form of calcium hydroxyapatite (HA) –Ca_10_(PO_4_)_6_(OH)_2_). The bone microenvironment and nanocomposites biocompatibility are highly preferred when bone repair nanoscale materials are selected or designed.

### Spongy bone and compact bone

There are two types of bone tissue present in most of the bones in the body; these are compact bone and spongy bone, representing 80% and 20% of the total bone mass, respectively.

Compact bone, is formed by cylindrical construction called Haversian or osteons systems and has an ordered histological pattern. The osteons run parallel to long bones and each of them contains lamellae that encircle a Harversian canal. Nerves and vessels go through the centric osteons canals whereas nutrients and waste products diffusion is limited. To exchange materials between the osteocytes and blood vessels, all the canaliculi build a branching network throughout compact bone. Based on the structural variations, bone regeneration materials should provide an adequate scaffold to support the autologous tissues, that is, shape and structure.

### Progress in bone fracture healing

Bone tissue repair and regeneration is a dynamic process that starts with proliferation and migration of osteoprogenitor cells, finally realizing the reconstruction of bone with differentiation of osteoprogenitor cells and bone ECM formation. The scaffold materials which load different growth factors and drugs have achieved a great progress in bone tissue engineering.^21^

BMPs are members of a TGF-β family^22,23^ that can significantly promote ossification in endochondral cells in mice after subcutaneous injection. Moreover, *in vivo* studies reported death of mice in early stages of development due to lack of BMP-2 or BMP-4.^24^

To date, even though the BMPs role in bone regeneration remains a challenge, some clinical studies give interesting clues. For instance, in fracture mouse model, the RNA levels of BMPs were tested during the course of damaged bone reparation. The results demonstrated that the BMP-2 and BMP-4 were expressed higher in early stages while BMP-5, BMP-6, and BMP-7 were expressed in terminal stages.^25^ In humans, the expression of BMP-2, BMP-3, BMP-4, and BMP-7 represents regional differences in a callus tissue, BMP-3 and BMP-7 by higher expression in generation of new osteoblasts while BMP-2 and BMP-4 mainly exist in the mature bone tissue or hypertrophic chondrocytes.^26^ All the clinical and pre-clinical researches support BMPs as an important factor in bone repair and clinical application.^27,28^

Wnt signaling molecules also have crucial role in regulating cell function, especially in osteogenesis and differentiation. Many Wnt proteins (for example, Wnt1, Wnt3a, Wnt4, Wnt5, Wnt10b and Wnt13) have key roles in regulating bone formation.^29^ The Wnt proteins can promote proliferation of mesenchymal stem cell (MSCs) and their osteogenic differentiation; however, it can also inhibit the formation of cartilage cells and fat cells.^30^ Zhong *et al.*^31^ found in rat models that, Wnt signaling members expressed significantly, both at transcription level and protein synthesis after bone defect occurred. Chen *et al.*^32^^,^^33^ found that fracture and β-catenin gene deletion in mice model can increase the proliferation of MSCs in the damage region, but they will differentiate to chondrocytes instead of osteoblasts, leading to the failure of bone repair. These results show that the Wnt/β-catenin signaling pathway is the core of mammalian bone biology and may provide new strategy for bone regeneration.^34^

The active factors that are loaded on scaffold materials, such as BMPs, Wnt, TGF-β, FGF, and VEGF can assemble the osteoprogenitor cells and induce them into specific cells, further regulating the regeneration of bone tissue and formation of ECM.^35^ *In vivo* studies also confirmed that the growth factor could enhance the reparation of various fractures (that is, cannot heal itself after certain period).^36^

### Requirements for materials in bone regeneration inflammation

Any material that has been utilized in bone repairs and as prosthesis should be highly acceptable to the biological system, with less adverse effects. In case of bone regeneration, various factors are affecting the healing process either that influence this process independently or as co-factor with other multiple factors. Mentioned below are some of the most pivotal properties of nanoscale biomaterials and composites for bone repair.

#### Biocompatibility

A perfect bone repair scaffold materials should neither suppress the activity of normal cells nor toxicity during and after implantation.^37^ In addition, it should also have osteogenesis-induced effects that may promote adhesion and proliferation of osteoblast or MSCs to form ECM. Cells can grow well in the three-dimensional (3D) microenvironment composed of nano-fibers. This is mainly because of a larger specific surface area that can promote adsorption of proteins, cell adhesion and growth.^38^ Meanwhile, more and more nanomaterials are being synthesized with good biocompatibility for biomedical applications.^39,40^

#### Mechanical property

The bone repair scaffold should satisfy the mechanical strength and provide transfer properties. Mechanical strength of bone tissue from cancellous to density has a broad range. The differences between mechanical strength and geometrical mechanics in bone tissue make it difficult to design an ideal scaffold.^41^ At the same time, various nanomaterials with good mechanical property are designed, such as nanofibers, nanopillars, nanoparticles, and nanocomposites,^42^ which may help in this challenge.

#### Vesicular structure

The vesicular structure is a necessity for bone repair scaffold materials with porous diameter in at least 100 μm, to ensure the transportation of nutrients and oxygen.^43^ It was found in one research study that scaffold materials with aperture size of 200–350 μm are best for bone tissue growth.^44^ In addition, recent studies have shown that the scaffold with multiple aperture sizes have better repair effect than materials with only large aperture.^45^ The bone repair materials have been successfully prepared by using polymer, ceramic, metal, and composite materials. Porous metal scaffolds can satisfy the mechanical requirements but cannot realize fusion with implants and tissues. Moreover, the metal ion will dissociate after implantation, which is also a serious problem that needs to be addressed.^46^

#### Bioabsorbability

Biological absorbability of the scaffold materials is another key factor for bone tissue regeneration.^37^ An ideal scaffold material should be degraded *in vivo* at a certain time, with a controllable absorption rate that will finally provide a space for new bone generation. The degradation time for scaffold should also satisfy the application requirements, such that the materials used in spinal fusion need to be degraded after 9 months or longer, whereas materials in skull or maxillofacial bone should degrade in 3–6 months. The nanoscale scaffold materials are porous and biodegradable that can also provide mechanical support during the bone repair.^47^

#### Angiogenesis

An important requirement for bone repair materials is to promote the angiogenesis due to higher blood demands in the bone tissues.^43,48–50^ The supply of oxygen and nutrients are indispensable for cells and tissues growth within the scaffold *in vivo.*^49^ The inflammatory reaction for wound healing can induce spontaneous formation of blood vessels after scaffold implant.^43^ It needs several weeks to form a vascular network; however, most scaffold materials do not have the ability to induce angiogenesis. In addition, incorrect or insufficient angiogenesis may hinder the delivery of oxygen and vital nutrients, which may result in uncontrolled differentiation or apoptosis of the cells.^51^

## Nanomaterials applied in bone repair and regeneration

The bone fracture, osteoporosis, osteoarthritis, and various neoplastic maladies are the most common clinical problems associated with bone and skeletal system. These common problems may be associated with malnutrition, aging, hormonal imbalance or trauma. It is estimated that around 2.2 million bone tissue graft transplants are performed all around the globe annually.^52^ The autograft is most common orthopedic implant but has certain well-documented limitations (that is, resorption, donor site morbidity, compromised supply, and rejection rate of up to 50% at some sites^53,54^). Mostly, the complicated and multiple fractures due to trauma or age (mostly at hip joint, that is, femur head fractures) are supported with prosthetic implants for proper healing. These implants are comprised of various materials known as biomaterials. Nevertheless, after 10–15 years on average, the traditional implant failure is associated with biomaterial associated inflammation, loosening, wear or tear debris, osteolysis and autoimmune reactions.^55^ These snags urge for the development of biomaterials with greater cytocompatibility and long lasting life, with higher patient’s quality of life. The role of nanotechnology and nanomaterials therefore becomes very pivotal. Various nanocomposites, materials and particles have been applied to mimic the growth of bone tissues, lower the autoimmune reactions and keep check on microbial infections.^56–59^ Herein, we also mainly focus on the nanomaterials role in bone tissue repair, support, and maintenance.

### Influence on bone regeneration

**Figure 1:**
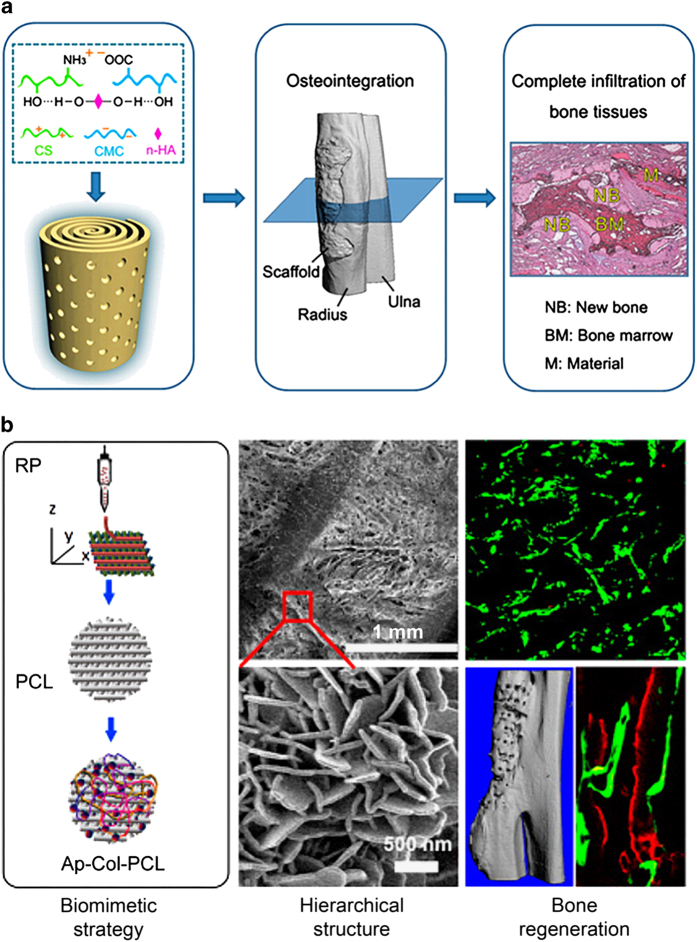
(**a**) Biomimetic spiral-cylindrical scaffold based on hybrid chitosan/cellulose/nano-hydroxyapatite membrane. (**b**) Biomimetically ornamented rapid prototyping fabrication of an apatite−collagen−polycaprolactone composite construct with nano−micro−macro hierarchical structure. Reprinted with permission from ref. 63 2013 ACS Publishing Group and ref. 68 2015 ACS Publishing Group.

Organic bone tissue has various protein (collagen, fibronectin, laminin, and vitronectin) and water as soft hydrogel nanocomposites, whereas HA and Ca_10_ (PO_4_)_6_ (OH)_2_ are hard inorganic components for the bone.^60^ The HA is present in nanocrystal line form which is 20–80 nm long and 2–5 nm thick, whereas the other proteins in the ECM are also at nanoscale size. This structural analogy allows the nanomaterials to interact easily with bone tissue and influence its functionality. Among the proposed nano-scaffolds for bone regeneration, Cerium (Ce-HA)^61^ based structures are among the leading candidates for bone tissue engineering. Similarly, a Mg-HA/collagen type I scaffold may also have great utility in bone regeneration.^62^ Besides, nano-HA together with chitosan (CS), Jiang *et al*.^63^ reported sodium carboxymethyl cellulose (CMC) hybrid membrane that was curled in a concentric manner to realize an anisotropic spiral–cylindrical scaffold. The cylinder-shaped scaffold has similarity to natural bone expedited complete infiltration of bone tissues *in vivo* and finally realized osteointegration and functional reconstruction of damage bone, as shown in Figure 1a.

Materials, at nanoscale, have been reported with better cell functionality than micro or macro scaled materials.^64^ The ECM provides scaffolds for the growth, proliferation and influence functionality of various cells. The nanoscale materials mimic the intrinsic and extrinsic pathways of osteocyte differentiation and mobility. Cells in various parts of the body exist in either two-dimensional (2D) or 3D environment, for example, stem cells in the intestinal crypts exists in 2D environment, whereas stem cells in bone marrow exist in 3D environment.^65^ The nanomaterials may provide the desired environment for the proliferation of various cells in a bone niche. Similarly, the magnetic nanoparticles in addition to influence on osteocytes intrinsic pathways, may also act as mechanical stimulus that will help in the healing process.^66^ The silk fibroin-hydroxybutyl chitosan blended nanofibers successfully provided scaffold for the growth of porcine iliac endothelial cells. The nanofibers provided typical ECM to cells, where these cells formed endothelial monolayer with higher confluency.^67^ In Figure 1b, Wang *et al.*^68^ developed apatite-collagen-polycaprolactone (Ap-Col-PCL) composites that showed excellent bioactivity to promote fast bone regeneration in rabbit model with fractional long bone defect. They combined rapid prototyping (RP) fabrication technology and 3D functionalization strategy for biomimetic deposition and collagen incorporation. These composite materials showed outstanding mechanical properties similar to cancellous bone, good biodegradability, and hierarchical architecture of three nano–micro–macro levels.^68^

### Bioactive materials

**Figure 2:**
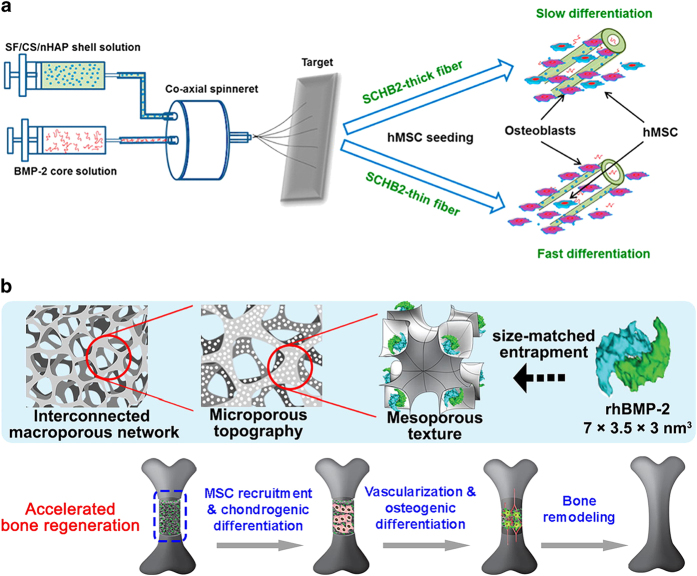
(**a**) Preparation of SCHB2-thick and SCHB2-thin NFMs through coaxial electrospinning and their influence on hMSCs. (**b**) Tri-modal macro/micro/nano-porous scaffold loaded with rhBMP-2 for accelerated bone regeneration. Reprinted with permission from ref. 76 2015 ACS Publishing Group and ref. 82 2016 Elsevier Publishing Group.

Bioactivity is the ability for a material to mimic response in living system.^69^ The orthopedic bioactive materials should elicit the biological response at interface and build a strong bond between the material and bone tissue.^70^ Hence, the role of bioactivity is inevitable for biomedical applications of biomaterials. The bioactive materials for bone repair are mainly divided into osteoconductive and osteoproductive, depending upon the rate of implant and its tissue interaction.^71^ The bioactive materials are mainly fabricated by either tailoring of bioactive composites and coatings or molecular surface tailoring. The later one is ideal for bone growth promoting factors, that is, BMPs. They are considered most important factors for the proliferation and growth of the bone tissue.^72^ The nanoarrays of gold has immobilizing effect on BMP-2, which allows the controlled release of BMP-2 that may have important role during the bone tissue repair via osteoblasts.^73,74^ The BMP-2 signals, differentiation and proliferation were also found to be significantly increased after treating cells with ceramic conjugated nanoparticles.^75^ Nanofibrous membranes (NFMs), for instance, with BMP-2 in the core and silk fibroin/chitosan/Nanohydroxyapatite (SCH) as the shell, were developed and tested both *in vitro* and *in vivo* for modulation of bone regeneration, results also suggesting the NFMs as an excellent scaffold for bone tissue engineering.^76^ (Figure 2a) Similarly, collagen-containing hydrogel was seeded with magnetic nanoparticles to target TWIK-related K(+) channel (TREK)-1 for enhanced mineralization on experimental basis. Moreover, the bone mineralization was significantly increased by mechano-transduction.^77^

#### Influence on bone tissue cells and BMSCs

In osteogenesis and bone mineralization studies, various biochemical mediators, including ascorbic acid, dexamethasone, BMPs, and β-glycerophosphate, are supplemented to differentiating medium or incorporated in biomaterials. However, some studies reported nanoscale composites with osteoinductive effect without addition of any biochemical mediator, suggesting strong influence of dimensional structure niche and cell’s shape.^78^ Notably, Khanna *et al*.^79^ reported chitosan-polygalactouronic acid hydroxyapatite (Chit-Pga-HA) nanofibers with osteoconductive and osteoinductive properties by mimicking the natural bone mineralization and collagen formation. Similarly, Roohani-Esfahani *et al.*^80^ coated biphasic calcium phosphate struts with bioactive glass nanoparticles and found 14 times increase in compressive strength and enhanced differentiation of primary human derived bone cells by upregulating the Runx2, osteopontin and sialoprotein genes. Moreover, the recent findings by Tutak *et al.*^81^ suggested that poly (ε-caprolactone) (PCL) nanofibers promoted the differentiation of human osteoprogenitor cells by changing the organelle structure and positioning, which resulted in altered cells functionality. Besides, as reported by Tang *et al.*,^82^ recombinant human bone morphogenetic protein-2 (rhBMP-2) was loaded on a tri-modal (macro/micro/nano) mesoporous bioactive glass scaffold (TMS) with enhanced compressive strength. They tailored a 7.5 nm, 3D cubic mesoporous structure for a “size-matched entrapment” of rhBMP-2, so the TMS/rhBMP-2 could achieve sustained release and appealing bone regeneration capacity (Figure 2b).

The nanostructure arrays of various biomaterials (for example, polymethylmethacrylate (PMMA) 120 nm pit and 100 nm diameter size with 300 nm interspace) have been reported with efficient osteogenic differentiation of bone marrow mesenchymal stem cells BMSCs.^83^ Similarly, Tarpani *et al.*^84^ used 130 nm silica (SiO_2_) nanoparticles functionalized by amino group (SiO_2_-N) and silver (SiO_2_-Ag) nanoparticles for the growth of human BMSCs and observed good interaction between the silica nanoparticles and BMSCs, making it a strong candidate for future bone tissue engineering. The recent findings by Rehman *et al.*^85^ also suggested strong proliferating effect of TiO_2_ nanowhiskers and tetra sulphonatophenyl porphyrin (TSPP) nanocomposites on rheumatoid arthritis BMSCs. In addition, Zuyaun *et al.*^86^ used the mesoporous silica based nanocomposites loaded with BMP-7 to differentiate the BMSC from osteocytes by slowly and constantly releasing the BMP-7 as trigger of the osteogenesis. Xia *et al.*^87^ reported the highly interconnected microporous HA bio ceramic scaffolds whose surface was modified by nanosheet, nano-rod and micro-nano-hybrids. The materials not only promoted cell attachment, proliferation, spreading and osteogenic differentiation of adipose derived stem cells (ASCs), but also enhanced the expression of angiogenic factors. The combination of the HA scaffolds with nano-surface and ASCs could enhance both osteogenesis and angiogenesis in a rat critical-sized calvarial defect model.^87^

#### Extra cellular matrix and bone supporting tissues

The bone tissue is a part of complex skeletal system that is also comprised of various tissues (for example, ligaments and tendons). The attached ligaments and tendons after trauma may also need regeneration; hence, the nanotechnology may be applied for enhancing strength and biocompatibility. Recently, Sheikh *et al.*^88^ incorporated multi-walled carbon nanotubes into polymeric nanofibers to form ideal candidate for bone tendon and ligament repair after trauma. They reported that addition of MWCNTs to the polymeric nanofibers increased the tensile strength from 11.40±0.9 to 51.25±5.5 Mpa.^88^ Moreover, the fibroblast cells attachment and higher viability rate indicated the biocompatibility of the said artificial ligaments/tendon candidate. The ligament advanced reinforcement system (LARS) is also considered a promising graft when nanomaterials, such as nano-silica, are applied to its surface; both the biocompatibility and ligament reconstruction effectiveness of LARS are improved.^89^ Moreover, some other studies reported the co-electrospun scaffold which was based on nano-hydroxyapatite particles, as well as Medtronic’s recombinant, could up-regulate the expression of BMP-2 and osteopontin on mineral-containing region, and may promote the regeneration of the ligament-bone interface.^90,91^

### Composite materials

Various synthesized hydrogels are good for providing extra cellular matrix for proliferation of cells during the healing process. The higher water content provides cells friendly microenvironment for performing various functions. Mostly during bone fracture the vasculature is compromised, which can be mimicked by various factors. The use of composite materials may allow the angiogenesis without any vital biochemical factor. The recent findings by Mammadov *et al.*^92^ suggest that the use of polymers to mimic angiogenesis without any soluble factor is a new approach in tissue regeneration. The same technique may be used for bone regeneration, especially in complex fractures where the vasculature is compromised.^92^ Other composite nanomaterials, such as Ca^2+^-induced Bombyx mori silk sericin (BS)/HA, reduced graphene oxide (rGO)/HA and recombinant human vascular endothelial growth factor (rhVEGF)/nano-HA/coralline blocks could also significantly promote the proliferation and osteogenic differentiation of the BMSCs for bone repair.^93–95^ Zhao *et al.*^96^ recently used tetra sulphonatophenyl porphyrin derivatives adjuvant with TiO_2_ nanowhiskers for theranostics of Rheumatoid Arthritis. These nanocomposites were not only biocompatible but also had protective effect on the synovial milieu and long bones tissue.^96^

Similarly, nano-TiO_2_ has been used for coating of orthopedic prosthetic implants.^97^ The TiO_2_ nanotubes have been used in the articular joints, that is, hip and knee joint, to minimize the wear and tear effect; however, it was not very successful due to inflammatory reactions. The nanoscale TiO_2_ particles coated on the surface of prosthetic implants are safer with enhanced bone mineralization and osteoblast adhesion.^98,99^ Earlier studies reported that orthopedic prosthetic implants coated with TiO_2_ nanotubes were successfully loaded with non-steroidal anti-inflammatory drugs (for example, Ibuprofen^100^) and variety of antibiotics and antibacterial (for example, gentamycin^101^ and cefuroxime^102^) to keep check on infection and inflammation, without compromising the adhesion of osteoblasts to the implanted biomaterial.

Bone tissue requires dynamic mechanical stimulation for its proper functionality. In nanotherapeutics, it can be fulfilled by various magnetic nanoparticles that upon exposure to magnetic field may alter the cells physiological and biochemical environment by moving the charged particles into the cell by enhanced membrane permeability.^64^

### Nano-coating of implants

Nanoscale structures and coating of various prosthetic implants is of higher interest in orthopedic surgery due to lower debris generation, especially in articular joints. The prosthesis main body is comprised of metallic alloy (that is, Ti-6AL-4 V, cobalt-chromium-molybdenum) which articulates against polymer or ceramic-polymer surface (alumina, aluminia-zirconia, ultra-high molecular weight PE). The excellent tribo-corosion and biocompatibility can be achieved via surface coating with nanotubes, nanowhiskers, diamond, and graphite like carbon, titanium,^103^ and tantalum.^104^ Along with anti-friction coating, the nanobiomaterials are also favored for control of infections by loading various antimicrobials on prosthesis surface. The nano-titania and silver particles coating on the prosthetic implants are very extensively used in orthopedic prosthetic implants to control post-operative complications and infections. Recently, Singh *et al.*^105^ prepared 25–35 nm HA coated on the Ti-alloy to lower the graft-versus-host disease (GVHD) to orthopedic implants and increased its biocompatibility. Stanic *et al.* synthesized Silver (Ag_2_O) fluroappatite nanopowder with 80 nm average length and 20 nm width, finding excellent antibacterial effect on klebsiella pneumoniae, Staphylococcus aureus and Micrococcus luteus due to the antibacterial effect of sliver, which can be potentially explored in orthopedic implants.^106^ Another biomimetic HA nano-construct was synthesized by Koirala *et al.*,^107^ which could modify a Ti implant. The nano-HA covered with a phospholipid bilayer may support long-term sustainability of implants. Although, the nanomaterials used in bone implants are also having adverse effects on the bone cells, for example, the silver (Ag) nanoparticles (80 nm) and ions are reported with delayed differentiation of human MSCs to osteocytes and adipocytes, even at biocompatible concentration.^108^

### 3D technology

**Figure 3:**
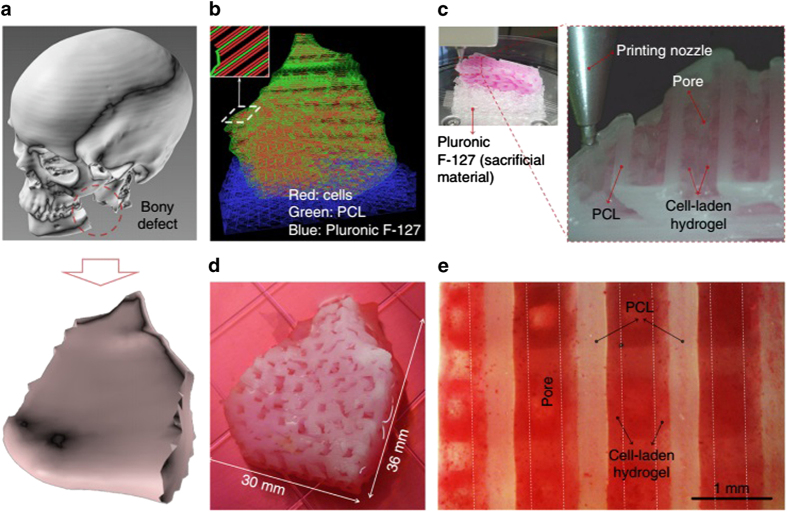
Mandible bone reconstruction. (**a**) 3D CAD model recognized a mandible bony defect from human CT image data. (**b**) Visualized motion program was generated to construct a 3D architecture of the mandible bone defect using CAM software. Lines of green, blue and red colors indicate the dispensing paths of PCL, Pluronic F127 and cell-laden hydrogel, respectively. (**c**) 3D printing process using integrated organ printing system. (**d**) Photograph of the 3D printed mandible bone defect construct, which was cultured in osteogenic medium for 28 days. (**e**) Osteogenic differentiation of hAFSCs in the printed construct was confirmed by Alizarin Red S staining, indicating calcium deposition. Reprinted with permission from ref. 112 2016 Nature Publishing Group.

In early eighties of twentieth century, Charles Hull was the first to report 3D technology for printing various objects. Afterwards, applications of the 3D technology got momentum in various fields, including biomedicine, for tissue regeneration and transplant, especially bone. Among various major concerns in the 3D technology, the bioresorption and biocompatibility are major issues. Most of the important bone materials used in bone 3D printing include calcium phosphate ceramics and cements, HA, brushite, monetite, β-tricalcium phosphate (TCP) and bioactive glass mixture, due to their comparable analogy to bone minerals and higher biocompatibility.^109,110^ Porosity of the implant is pivotal for the growth and attachment of bone tissue to implant. The ideal porosity has been reported with 30%–70% prosthetic comprised of 500–1000 μm, respectively.^111^ However, the bone tissue is comprised of various nano (collagen– I and other proteins) to macro structures, and subtle disturbance or disorientation in these structures will lead to maladies (for example, osteogenesis imperfecta and brittle bone disease). Recently, Kang *et al.*^112^ demonstrated a mandible bone reconstruction using human amniotic fluid–derived stem cell (hAFSC)-laden hydrogel, a mixture of PCL and TCP, and Pluronic F127 (Figure 3b). The PCL/TCP and hAFSCs mixed with the composite hydrogel were printed in a type I pattern with a Pluronic F127 temporary support (Figure 3c). After induction of osteogenic differentiation for 28 days (Figure 3d), they stained the structures with Alizarin Red S; staining at the surface of the 3D bone structures indicated calcium deposition in the hAFSC-laden hydrogel (Figure 3e).

The HA based nanocomposites are favored for the nano 3D structure formation due to promotion of cell organization, proliferation and allowance of free movement of nutrients to the developing tissues. The recent findings by Jun *et al.* suggest that sphere shaped nano-HA-chitosan-gelatin based scaffolds accelerated the fibroblast iPSC (induced pluripotent stem cells) osteogenesis as compared with rod shaped nano-HA-chitosan-gelatin scaffold both *in vivo* and *in vitro.*^113^

## Conclusion

In summary, biomedical applications of the nanoscale materials in amelioration and regeneration of skeletal system, especially in bone and supporting tissues, are highly appreciated in modern therapeutics and surgery. The 3D scaffold, structural analogy, biocompatibility, growth promoting properties, and time-bound degradability of nanoscale materials make them ideal candidates for orthopedic prosthetic surgeries and bone reparation.

## References

[bib1] Brooks PM. The burden of musculoskeletal disease—a global perspective. Clin Rheumatol 2006; 25: 778–781. 16609823 10.1007/s10067-006-0240-3

[bib2] Collaborators GDaH. Global, regional, and national disability-adjusted life years (DALYs) for 306 diseases and injuries and healthy life expectancy (HALE) for 188 countries, 1990–2013: quantifying the epidemiological transition. Lancet 2015; 386: 2145–2191. 26321261 10.1016/S0140-6736(15)61340-X PMC4673910

[bib3] Salomon JA, Vos T, Hogan DR et al. Common values in assessing health outcomes from disease and injury: disability weights measurement study for the Global Burden of Disease Study 2010. Lancet 2012; 380: 2129–2143. 23245605 10.1016/S0140-6736(12)61680-8 PMC10782811

[bib4] Mouriño V, Boccaccini AR. Bone tissue engineering therapeutics: controlled drug delivery in three-dimensional scaffolds. J R Soc Interface 2010; 7: 209–227. 19864265 10.1098/rsif.2009.0379 PMC2842615

[bib5] Wahajuddin, Arora S. Superparamagnetic iron oxide nanoparticles: magnetic nanoplatforms as drug carriers. Int J Nanomedicine 2012; 7: 3445–3471. 22848170 10.2147/IJN.S30320 PMC3405876

[bib6] Fernández-Urrusuno R, Fattal E, Rodrigues JM Jr et al. Effect of polymeric nanoparticle administration on the clearance activity of the mononuclear phagocyte system in mice. J Biomed Mater Res 1996; 31: 401–408. 8806067 10.1002/(SICI)1097-4636(199607)31:3<401::AID-JBM15>3.0.CO;2-L

[bib7] Katz E, Willner I. Integrated nanoparticle-biomolecule hybrid systems: synthesis, properties, and applications. Angew Chem Int Ed Engl 2004; 43: 6042–6108. 15538757 10.1002/anie.200400651

[bib8] Shah MA, He N, Li Z et al. Nanoparticles for DNA vaccine delivery. J Biomed Nanotechnol 2014; 10: 2332–2349. 25992460 10.1166/jbn.2014.1981

[bib9] Mou X, Ali Z, Li S et al. Applications of magnetic nanoparticles in targeted drug delivery system. J Nanosci Nanotechnol 2015; 15: 54–62. 26328305 10.1166/jnn.2015.9585

[bib10] Liu M, Hu P, Zhang G et al. Copy number variation analysis by ligation-dependent PCR based on magnetic nanoparticles and chemiluminescence. Theranostics 2015; 5: 71–85. 25553099 10.7150/thno.10117 PMC4265749

[bib11] He N, Wang F, Ma C et al. Chemiluminescence analysis for HBV-DNA hybridization detection with magnetic nanoparticles based DNA extraction from positive whole blood samples. J Biomed Nanotechnol 2013; 9: 267–273. 23627053 10.1166/jbn.2013.1478

[bib12] Xi Z, Huang R, Li Z et al. Selection of HBsAg-specific DNA aptamers based on carboxylated magnetic nanoparticles and their application in the rapid and simple detection of hepatitis B virus infection. ACS Appl Mater Interfaces 2015; 7: 11215–11223. 25970703 10.1021/acsami.5b01180

[bib13] Huang R, Xi Z, He N. Applications of aptamers for chemistry analysis, medicine and food security. Sci China Chem 2015; 58: 1122–1130.

[bib14] Wang J, Ali Z, Wang N et al. Simultaneous extraction of DNA and RNA from Escherichia coli BL 21 based on silica-coated magnetic nanoparticles. Sci China Chem 2015; 58: 1774–1778.

[bib15] Zhao C, Ur Rehman F, Jiang H et al. Titanium dioxide-tetra sulphonatophenyl porphyrin nanocomposites for target cellular bio-imaging and treatment of rheumatoid arthritis. Sci China Chem 2016; 59: 637–642.

[bib16] Yao C, Webster TJ. Anodization: a promising nano-modification technique of titanium implants for orthopedic applications. J Nanosci Nanotechnol 2006; 6: 2682–2692. 17048475 10.1166/jnn.2006.447

[bib17] Rehman FU, Zhao C, Jiang H et al. Biomedical applications of nano-titania in theranostics and photodynamic therapy. Biomater Sci 2016; 4: 40–54. 26442645 10.1039/c5bm00332f

[bib18] Liao J, Shi K, Ding Q et al. Recent developments in scaffold-guided cartilage tissue regeneration. J Biomed Nanotechnol 2014; 10: 3085–3104. 25992430 10.1166/jbn.2014.1934

[bib19] Fuchs R, Warden S, Turner C. Bone anatomy, physiology and adaptation to mechanical loading. In: Planell JA, Best SM, Lacroix D Bone Repair Biomaterials. Cambridge: Woodhead Publishing Limited and CRC Press LLC, 2009: 25.

[bib20] Gordon JA, Tye CE, Sampaio AV et al. Bone sialoprotein expression enhances osteoblast differentiation and matrix mineralization *in vitro*. Bone 2007; 41: 462–473. 17572166 10.1016/j.bone.2007.04.191

[bib21] Kumar JP, Lakshmi L, Jyothsna V et al. Synthesis and characterization of diopside particles and their suitability along with chitosan matrix for bone tissue engineering *in vitro* and *in vivo*. J Biomed Nanotechnol 2014; 10: 970–981. 24749392 10.1166/jbn.2014.1808

[bib22] Grimaud E, Heymann D, Redini F. Recent advances in TGF-beta effects on chondrocyte metabolism. Potential therapeutic roles of TGF-beta in cartilage disorders. Cytokine Growth Factor Rev 2002; 13: 241–257. 12486877 10.1016/s1359-6101(02)00004-7

[bib23] Chen D, Zhao M, Mundy GR. Bone morphogenetic proteins. Growth Factors 2004; 22: 233–241. 15621726 10.1080/08977190412331279890

[bib24] Gimble JM, Morgan C, Kelly K et al. Bone morphogenetic proteins inhibit adipocyte differentiation by bone marrow stromal cells. J Cell Biochem 1995; 58: 393–402. 7593260 10.1002/jcb.240580312

[bib25] Dimitriou R, Tsiridis E, Giannoudis PV. Current concepts of molecular aspects of bone healing. Injury 2005; 36: 1392–1404. 16102764 10.1016/j.injury.2005.07.019

[bib26] Kloen P, Di Paola M, Borens O et al. BMP signaling components are expressed in human fracture callus. Bone 2003; 33: 362–371. 13678778 10.1016/s8756-3282(03)00191-1

[bib27] Axelrad TW, Einhorn TA. Bone morphogenetic proteins in orthopaedic surgery. Cytokine Growth Factor Rev 2009; 20: 481–488. 19892584 10.1016/j.cytogfr.2009.10.003

[bib28] Rodríguez-Evora M, Reyes R, Alvarez-Lorenzo C et al. Bone regeneration induced by an *in situ* gel-forming poloxamine, bone morphogenetic protein-2 system. J Biomed Nanotechnol 2014; 10: 959–969. 24749391 10.1166/jbn.2014.1801

[bib29] Liu F, Kohlmeier S, Wang CY. Wnt signaling and skeletal development. Cell Signal 2008; 20: 999–1009. 18164181 10.1016/j.cellsig.2007.11 PMC2413267

[bib30] Takada I, Mihara M, Suzawa M et al. A histone lysine methyltransferase activated by non-canonical Wnt signalling suppresses PPAR-gamma transactivation. Nat Cell Biol 2007; 9: 1273–1285. 17952062 10.1038/ncb1647

[bib31] Zhong N, Gersch RP, Hadjiargyrou M. Wnt signaling activation during bone regeneration and the role of Dishevelled in chondrocyte proliferation and differentiation. Bone 2006; 39: 5–16. 16459154 10.1016/j.bone.2005.12.008

[bib32] Chen Y, Whetstone HC, Lin AC et al. Beta-catenin signaling plays a disparate role in different phases of fracture repair: implications for therapy to improve bone healing. PLoS Med 2007; 4: e249. 17676991 10.1371/journal.pmed.0040249 PMC1950214

[bib33] Chen Y, Whetstone HC, Youn A et al. Beta-catenin signaling pathway is crucial for bone morphogenetic protein 2 to induce new bone formation. J Biol Chem 2007; 282: 526–533. 17085452 10.1074/jbc.M602700200

[bib34] Yadav VK, Ryu JH, Suda N et al. Lrp5 controls bone formation by inhibiting serotonin synthesis in the duodenum. Cell 2008; 135: 825–837. 19041748 10.1016/j.cell.2008.09.059 PMC2614332

[bib35] Cao H, Kuboyama N. A biodegradable porous composite scaffold of PGA/beta-TCP for bone tissue engineering. Bone 2010; 46: 386–395. 19800045 10.1016/j.bone.2009.09.031

[bib36] Li J, Hong J, Zheng Q et al. Repair of rat cranial bone defects with nHAC/PLLA and BMP-2-related peptide or rhBMP-2. J Orthop Res 2011; 29: 1745–1752. 21500252 10.1002/jor.21439

[bib37] Williams DF. On the mechanisms of biocompatibility. Biomaterials 2008; 29: 2941–2953. 18440630 10.1016/j.biomaterials.2008.04.023

[bib38] Stevens MM, George JH. Exploring and engineering the cell surface interface. Science 2005; 310: 1135–1138. 16293749 10.1126/science.1106587

[bib39] Chen D, Gao S, Ur Rehman F et al. *In-situ* green synthesis of highly active GSH-capped Pt-Au-Ag-hybrid nanoclusters. Sci China Chem 2014; 57: 1532–1537.

[bib40] Rehman FU, Zhao C, Jiang H et al. Biomedical applications of nano-titania in theranostics and photodynamic therapy. Biomater Sci 2016; 4: 40–54. 26442645 10.1039/c5bm00332f

[bib41] Bowman BM, Siska CC, Miller SC. Greatly increased cancellous bone formation with rapid improvements in bone structure in the rat maternal skeleton after lactation. J Bone Miner Res 2002; 17: 1954–1960. 12412802 10.1359/jbmr.2002.17.11.1954

[bib42] Aston DE, Bow JR, Gangadean DN. Mechanical properties of selected nanostructured materials and complex bio-nano, hybrid and hierarchical systems. Int Mater Rev 2013; 58: 167–202.

[bib43] Rouwkema J, Rivron NC, van Blitterswijk CA. Vascularization in tissue engineering. Trends Biotechnol 2008; 26: 434–441. 18585808 10.1016/j.tibtech.2008.04.009

[bib44] Murphy CM, Haugh MG, O'Brien FJ. The effect of mean pore size on cell attachment, proliferation and migration in collagen-glycosaminoglycan scaffolds for bone tissue engineering. Biomaterials 2010; 31: 461–466. 19819008 10.1016/j.biomaterials.2009.09.063

[bib45] Woodard JR, Hilldore AJ, Lan SK et al. The mechanical properties and osteoconductivity of hydroxyapatite bone scaffolds with multi-scale porosity. Biomaterials 2007; 28: 45–54. 16963118 10.1016/j.biomaterials.2006.08.021

[bib46] Rezwan K, Chen QZ, Blaker JJ et al. Biodegradable and bioactive porous polymer/inorganic composite scaffolds for bone tissue qengineering. Biomaterials 2006; 27: 3413–3431. 16504284 10.1016/j.biomaterials.2006.01.039

[bib47] Khan Y, Yaszemski MJ, Mikos AG et al. Tissue engineering of bone: material and matrix considerations. J Bone Joint Surg Am 2008; 90: 36–42. 18292355 10.2106/JBJS.G.01260

[bib48] Bramfeld H, Sabra G, Centis V et al. Scaffold vascularization: a challenge for three-dimensional tissue engineering. Curr Med Chem 2010; 17: 3944–3967. 20939827 10.2174/092986710793205327

[bib49] Jain RK, Au P, Tam J et al. Engineering vascularized tissue. Nat Biotechnol 2005; 23: 821–823. 16003365 10.1038/nbt0705-821

[bib50] Tomlinson RE, Silva MJ. Skeletal Blood Flow in Bone Repair and Maintenance. Bone Res 2013; 1: 311–322. 26273509 10.4248/BR201304002 PMC4472118

[bib51] Malda J, Rouwkema J, Martens DE et al. Oxygen gradients in tissue-engineered PEGT/PBT cartilaginous constructs: measurement and modeling. Biotechnol Bioeng 2004; 86: 9–18. 15007836 10.1002/bit.20038

[bib52] Giannoudis PV, Dinopoulos H, Tsiridis E. Bone substitutes: an update. Injury 2005; 36: S20–S27. 16188545 10.1016/j.injury.2005.07.029

[bib53] Bajaj AK, Wongworawat AA, Punjabi A. Management of alveolar clefts. J Craniofac Surg 2003; 14: 840–846. 14600625 10.1097/00001665-200311000-00005

[bib54] Clavero J, Lundgren S. Ramus or chin grafts for maxillary sinus inlay and local onlay augmentation: comparison of donor site morbidity and complications. Clin Implant Dent Relat Res 2003; 5: 154–160. 14575631 10.1111/j.1708-8208.2003.tb00197.x

[bib55] Zhang L, Webster TJ. Nanotechnology and nanomaterials: Promises for improved tissue regeneration. Nano Today 2009; 4: 66–80.

[bib56] Liu Y, Luo D, Liu S et al. Effect of nanostructure of mineralized collagen scaffolds on their physical properties and osteogenic potential. J Biomed Nanotechnol 2014; 10: 1049–1060. 24749399 10.1166/jbn.2014.1794

[bib57] Cheng Y, Ramos D, Lee P et al. Collagen functionalized bioactive nanofiber matrices for osteogenic differentiation of mesenchymal stem cells: bone tissue engineering. J Biomed Nanotechnol 2014; 10: 287–298. 24738337 10.1166/jbn.2014.1753

[bib58] Fu S, Ni P, Wang B et al. *In vivo* biocompatibility and osteogenesis of electrospun poly(epsilon-caprolactone)-poly(ethylene glycol)-poly(epsilon-caprolactone)/nano-hydroxyapatite composite scaffold. Biomaterials 2012; 33: 8363–8371. 22921926 10.1016/j.biomaterials.2012.08.023

[bib59] Fu S, Ni P, Wang B et al. Injectable and thermo-sensitive PEG-PCL-PEG copolymer/collagen/n-HA hydrogel composite for guided bone regeneration. Biomaterials 2012; 33: 4801–4809. 22463934 10.1016/j.biomaterials.2012.03.040

[bib60] Basu B, Katti DS, Kumar A. Advanced Biomaterials: Fundamentals, Processing, and Applications. Hoboken: John Wiley & Sons, 2010.

[bib61] Sun LJ, Guo DG, Zhao WA et al. Influences of reaction parameters and Ce contents on structure and properties of nano-scale Ce-HA powders. J Mater Sci Technol 2014; 30: 776–781.

[bib62] Minardi S, Corradetti B, Taraballi F et al. Evaluation of the osteoinductive potential of a bio-inspired scaffold mimicking the osteogenic niche for bone augmentation. Biomaterials 2015; 62: 128–137. 26048479 10.1016/j.biomaterials.2015.05.011

[bib63] Jiang H, Zuo Y, Zou Q et al. Biomimetic spiral-cylindrical scaffold based on hybrid chitosan/cellulose/nano-hydroxyapatite membrane for bone regeneration. ACS Appl Mater Interfaces 2013; 5: 12036–12044. 24191736 10.1021/am4038432

[bib64] Balasundaram G, Storey DM, Webster TJ. Novel nano-rough polymers for cartilage tissue engineering. Int J Nanomedicine 2014; 9: 1845–1853. 24790427 10.2147/IJN.S55865 PMC3998868

[bib65] Turner L, Dalby MJ. Nanotopography–potential relevance in the stem cell niche. Biomater Sci 2014; 2: 1574–1594. 10.1039/c4bm00155a 32481943

[bib66] Wang Q, Yan J, Yang J et al. Nanomaterials promise better bone repair. Mater Today 2016; 19: 451–463.

[bib67] Zhang K, Qian Y, Wang H et al. Electrospun silk fibroin-hydroxybutyl chitosan nanofibrous scaffolds to biomimic extracellular matrix. J Biomater Sci Polym Ed 2011; 22: 1069–1082. 20615313 10.1163/092050610X498204

[bib68] Wang J, Wu D, Zhang Z et al. Biomimetically ornamented rapid prototyping fabrication of an apatite-collagen-polycaprolactone composite construct with nano-micro-macro hierarchical structure for large bone defect treatment. ACS Appl Mater Interfaces 2015; 7: 26244–26256. 26551161 10.1021/acsami.5b08534

[bib69] Grotra D, Subbarao CV. Bioactive materials used in endodontics. Rec Res Sci Technol 2012; 4: 25–27.

[bib70] Gandolfi M, Taddei P, Tinti A et al. Apatite‐forming ability (bioactivity) of ProRoot MTA. Int Endod J 2010; 43: 917–929. 20646080 10.1111/j.1365-2591.2010.01768.x

[bib71] Cao W, Hench LL. Bioactive materials. Ceram Int 1996; 22: 493–507.

[bib72] Liang K, Li XC, Tay BK. Study of bone morphogenetic protein-2 delivery with different TiO2 nanotube structures. Nanosci Nanotechnol Lett 2013; 5: 162–166.

[bib73] Luginbuehl V, Meinel L, Merkle HP et al. Localized delivery of growth factors for bone repair. Eur J Pharm Biopharm 2004; 58: 197–208. 15296949 10.1016/j.ejpb.2004.03.004

[bib74] Schwab EH, Pohl TL, Haraszti T et al. Nanoscale control of surface immobilized BMP-2: toward a quantitative assessment of BMP-mediated signaling events. Nano Lett 2015; 15: 1526–1534. 25668064 10.1021/acs.nanolett.5b00315

[bib75] Wu Y, Jiang W, Wen X et al. A novel calcium phosphate ceramic-magnetic nanoparticle composite as a potential bone substitute. Biomed Mater 2010; 5: 15001. 20057017 10.1088/1748-6041/5/1/015001

[bib76] Shalumon KT, Lai GJ, Chen CH et al. Modulation of bone-specific tissue regeneration by incorporating bone morphogenetic protein and controlling the shell thickness of silk fibroin/chitosan/nanohydroxyapatite core-shell nanofibrous membranes. ACS Appl Mater Interfaces 2015; 7: 21170–21181. 26355766 10.1021/acsami.5b04962

[bib77] Henstock JR, Rotherham M, Rashidi H et al. Remotely activated mechanotransduction via magnetic nanoparticles promotes mineralization synergistically with bone morphogenetic protein 2: applications for injectable cell therapy. Stem Cells Transl Med 2014; 3: 1363–1374. 25246698 10.5966/sctm.2014-0017 PMC4214839

[bib78] Ribeiro A, Vargo S, Powell EM et al. Substrate three-dimensionality induces elemental morphological transformation of sensory neurons on a physiologic timescale. Tissue Eng Part A 2012; 18: 93–102. 21910606 10.1089/ten.tea.2011.0221 PMC3246411

[bib79] Khanna R, Katti KS, Katti DR. Bone nodules on chitosan-polygalacturonic acid-hydroxyapatite nanocomposite films mimic hierarchy of natural bone. Acta Biomater 2011; 7: 1173–1183. 21034863 10.1016/j.actbio.2010.10.028

[bib80] Roohani-Esfahani SI, Nouri-Khorasani S, Lu ZF et al. Effects of bioactive glass nanoparticles on the mechanical and biological behavior of composite coated scaffolds. Acta Biomater 2011; 7: 1307–1318. 20971219 10.1016/j.actbio.2010.10.015

[bib81] Tutak W, Jyotsnendu G, Bajcsy P et al. Nanofiber scaffolds influence organelle structure and function in bone marrow stromal cells. J Biomed Mater Res B Appl Biomater 2016; doi: 10.1002/jbm.b.33624. [Epub ahead of print]. 10.1002/jbm.b.33624 PMC6349397 26888543

[bib82] Tang W, Lin D, Yu Y et al. Bioinspired trimodal macro/micro/nano-porous scaffolds loading rhBMP-2 for complete regeneration of critical size bone defect. Acta Biomater 2016; 32: 309–323. 26689464 10.1016/j.actbio.2015.12.006

[bib83] Dalby MJ, Gadegaard N, Tare R et al. The control of human mesenchymal cell differentiation using nanoscale symmetry and disorder. Nat Mater 2007; 6: 997–1003. 17891143 10.1038/nmat2013

[bib84] Tarpani L, Morena F, Gambucci M et al. The Influence of Modified Silica Nanomaterials on Adult Stem Cell Culture. Nanomaterials 2016; 6: 104–114. 10.3390/nano6060104 PMC5302627 28335232

[bib85] Rehman FU, Zhao C, Wu C et al. Influence of photoactivated tetra sulphonatophenyl porphyrin and TiO2nanowhiskers on rheumatoid arthritis infected bone marrow stem cell proliferation *in vitro* and oxidative stress biomarkers *in vivo*. RSC Adv 2015; 5: 107285–107292.

[bib86] Luo Z, Deng Y, Zhang R et al. Peptide-laden mesoporous silica nanoparticles with promoted bioactivity and osteo-differentiation ability for bone tissue engineering. Colloids Surf B Biointerfaces 2015; 131: 73–82. 25969416 10.1016/j.colsurfb.2015.04.043

[bib87] Xia L, Lin K, Jiang X et al. Effect of nano-structured bioceramic surface on osteogenic differentiation of adipose derived stem cells. Biomaterials 2014; 35: 8514–8527. 25002263 10.1016/j.biomaterials.2014.06.028

[bib88] Sheikh FA, Macossay J, Cantu T et al. Imaging, spectroscopy, mechanical, alignment and biocompatibility studies of electrospun medical grade polyurethane (Carbothane 3575A) nanofibers and composite nanofibers containing multiwalled carbon nanotubes. J Mech Behav Biomed Mater 2015; 41: 189–198. 25460415 10.1016/j.jmbbm.2014.10.012 PMC4312223

[bib89] Li M, Wang S, Jiang J et al. Surface modification of nano-silica on the ligament advanced reinforcement system for accelerated bone formation: primary human osteoblasts testing *in vitro* and animal testing *in vivo*. Nanoscale 2015; 7: 8071–8075. 25872493 10.1039/c5nr01439e

[bib90] Mariner PD, Wudel JM, Miller DE et al. Synthetic hydrogel scaffold is an effective vehicle for delivery of INFUSE (rhBMP2) to critical-sized calvaria bone defects in rats. J Orthop Res 2013; 31: 401–406. 23070779 10.1002/jor.22243 PMC3565235

[bib91] Samavedi S, Guelcher SA, Goldstein AS et al. Response of bone marrow stromal cells to graded co-electrospun scaffolds and its implications for engineering the ligament-bone interface. Biomaterials 2012; 33: 7727–7735. 22835644 10.1016/j.biomaterials.2012.07.008

[bib92] Mammadov R, Mammadov B, Toksoz S et al. Heparin mimetic peptide nanofibers promote angiogenesis. Biomacromolecules 2011; 12: 3508–3519. 21853983 10.1021/bm200957s

[bib93] Lee JH, Shin YC, Lee SM et al. Enhanced osteogenesis by reduced graphene oxide/hydroxyapatite nanocomposites. Sci Rep 2015; 5: 18833. 26685901 10.1038/srep18833 PMC4685392

[bib94] Du B, Liu W, Deng Y et al. Angiogenesis and bone regeneration of porous nano-hydroxyapatite/coralline blocks coated with rhVEGF165 in critical-size alveolar bone defects *in vivo*. Int J Nanomedicine 2015; 10: 2555–2565. 25848271 10.2147/IJN.S78331 PMC4386782

[bib95] Yang M, Zhou G, Shuai Y et al. Ca^2+^-induced self-assembly of Bombyx mori silk sericin into a nanofibrous network-like protein matrix for directing controlled nucleation of hydroxylapatite nano-needles. J Mater Chem B Mater Biol Med 2015; 3: 2455–2462. 26029374 10.1039/C4TB01944J PMC4449145

[bib96] Zhao C, Ur Rehman F, Yang Y et al. Bio-imaging and photodynamic therapy with tetra sulphonatophenyl porphyrin (TSPP)-TiO2 nanowhiskers: new approaches in rheumatoid arthritis theranostics. Sci Rep 2015; 5: 11518. 26153895 10.1038/srep11518 PMC4648397

[bib97] Wang JX, Xu SB, Cheng SF et al. Enhanced photocatalytic properties of hierarchical microstructured TiO2 spheres synthesized with titanium powders. Nanosci Nanotechnol Lett 2015; 7: 252–256.

[bib98] Jain S, Jain AP, Jain S et al. Nanotechnology: an emerging area in the field of dentistry. J Dent Sci 2013; http://dx.doi.org/10.1016/j.jds.2013.08.004.

[bib99] Escada A, Nakazato R, Claro A. Growth of TiO_2_ nanotubes by anodization of Ti–7.5 Mo in NH_4_F solutions. Nanosci Nanotechnol Lett 2013; 5: 510–512.

[bib100] Zhao P, Liu H, Deng H et al. A study of chitosan hydrogel with embedded mesoporous silica nanoparticles loaded by ibuprofen as a dual stimuli-responsive drug release system for surface coating of titanium implants. Colloids Surf B Biointerfaces 2014; 123: 657–663. 25456989 10.1016/j.colsurfb.2014.10.013

[bib101] Chennell P, Feschet-Chassot E, Devers T et al. *In vitro* evaluation of TiO_2_ nanotubes as cefuroxime carriers on orthopaedic implants for the prevention of periprosthetic joint infections. Int J Pharm 2013; 455: 298–305. 23892151 10.1016/j.ijpharm.2013.07.014

[bib102] Pérez-Anes A, Gargouri M, Laure W et al. Bioinspired titanium drug eluting platforms based on a poly-beta-cyclodextrin-chitosan layer-by-layer self-assembly targeting infections. ACS Appl Mater Interfaces 2015; 7: 12882–12893. 25992843 10.1021/acsami.5b02402

[bib103] Yun KD, Park SW, Lee KM et al. Titanium dioxide nanotube modified implants: an animal study on bone formation. J Nanosci Nanotechnol 2013; 13: 3864–3867. 23862419 10.1166/jnn.2013.7023

[bib104] Ching HA, Choudhury D, Nine MJ et al. Effects of surface coating on reducing friction and wear of orthopaedic implants. Sci Technol Adv Mater 2014; 15: 014402. 27877638 10.1088/1468-6996/15/1/014402 PMC5090599

[bib105] Singh S, Meena VK, Sharma M et al. Preparation and coating of nano-ceramic on orthopaedic implant material using electrostatic spray deposition. Mater Des 2015; 88: 278–286.

[bib106] Stanić V, Radosavljević-Mihajlović AS, Živković-Radovanović V et al. Synthesis, structural characterisation and antibacterial activity of Ag^+^-doped fluorapatite nanomaterials prepared by neutralizationmethod. Appl Surf Sci 2015; 337: 72–80.

[bib107] Koirala MB, Nguyen TD, Pitchaimani A et al. Synthesis and characterization of biomimetic hydroxyapatite nanoconstruct using chemical gradient across lipid bilayer. ACS Appl Mater Interfaces 2015; 7: 27382–27390. 26574639 10.1021/acsami.5b09042

[bib108] Sengstock C, Diendorf J, Epple M et al. Effect of silver nanoparticles on human mesenchymal stem cell differentiation. Beilstein J Nanotechnol 2014; 5: 2058–2069. 25551033 10.3762/bjnano.5.214 PMC4273214

[bib109] Tadic D, Epple M. A thorough physicochemical characterisation of 14 calcium phosphate-based bone substitution materials in comparison to natural bone. Biomaterials 2004; 25: 987–994. 14615163 10.1016/s0142-9612(03)00621-5

[bib110] Bergmann C, Lindner M, Zhang W et al. 3D printing of bone substitute implants using calcium phosphate and bioactive glasses. J Eur Ceram Soc 2010; 30: 2563–2567.

[bib111] Curodeau A, Sachs E, Caldarise S. Design and fabrication of cast orthopedic implants with freeform surface textures from 3-D printed ceramic shell. J Biomed Mater Res 2000; 53: 525–535. 10984701 10.1002/1097-4636(200009)53:5<525::aid-jbm12>3.0.co;2-1

[bib112] Kang HW, Lee SJ, Ko IK et al. A 3D bioprinting system to produce human-scale tissue constructs with structural integrity. Nat Biotechnol 2016; 34: 312–319. 26878319 10.1038/nbt.3413

[bib113] Ji J, Tong X, Huang X et al. Sphere-shaped nano-hydroxyapatite/chitosan/gelatin 3D porous scaffolds increase proliferation and osteogenic differentiation of human induced pluripotent stem cells from gingival fibroblasts. Biomed Mater 2015; 10: 045005. 26154827 10.1088/1748-6041/10/4/045005

